# Herding Friends in Similarity-Based Architecture of Social Networks

**DOI:** 10.1038/s41598-020-61330-6

**Published:** 2020-03-17

**Authors:** Tamas David-Barrett

**Affiliations:** 1 Universidad del Desarrollo, Facultad de Gobierno, CICS, Av. Plaza 680, San Carlos de Apoquindo, Las Condes, Santiago de Chile 7610658 Chile; 2 University of Oxford, South Parks Road, Oxford, OX1 3UD United Kingdom; 3 Population Research Institute, Väestöliitto, Kalevankatu 16, Helsinki, 00101 Finland

**Keywords:** Behavioural ecology, Human behaviour

## Abstract

Although friendship as a social behaviour is an evolved trait that shares many similarities with kinship, there is a key difference: to choose friends, one must select few from many. Homophily, i.e., a similarity-based friendship choice heuristic, has been shown to be the main factor in selecting friends. Its function has been associated with the efficiency of collective action via synchronised mental states. Recent empirical results question the general validity of this explanation. Here I offer an alternative hypothesis: similarity-based friendship choice is an individual-level adaptive response to falling clustering coefficient of the social network typical during urbanisation, falling fertility, increased migration. The mathematical model shows how homophily as a friend-choice heuristic affects the network structure: (1) homophilic friendship choice increases the clustering coefficient; (2) network proximity-based and similarity-based friendship choices have additive effects on the clustering coefficient; and (3) societies that face falling fertility, urbanisation, and migration, are likely go through a u-shaped transition period in terms of clustering coefficient. These findings suggest that social identity can be seen as an emergent phenomenon and is the consequence, rather than the driver of, homophilic social dynamics, and offer an alternative explanation for the rise of “fake news” as a societal phenomenon.

## Introduction

Friendship is prevalent in all cultures: people in every society habitually create long-lasting social bonds with non-kin outside the mating context^1–5^. For chimpanzees, although kin-relationships are the primary norm, non-kin friendship is frequent both in within-sex female^6^ and male dyads^7^. The fact that friendship formation behaviour is universal in humans and appears in similar form in our species’ close relatives points towards evolutionary roots^8–11^.

Although friendship is similar to kinship in many ways^12^, there is one key difference: friendship is always more of a choice than kinship. While our family tends to be pre-determined^13,14^, we can and do choose our friends. The propensity to make such non-kin, non-mating positive social affiliations raises a practical problem: who to make friends with? This is an important question: friendship can be a long-lasting bond that requires substantial investment both to create and to maintain^4,15^.

Both humans^16–18^ and chimpanzees^10^ use trait similarity as the primary friendship choice heuristics. Why is it similarity rather than compatible difference that drives friendship choice? Arguably, the latter would allow division of labour through specialisation. The literature’s existing argument^19^ is that homophily increases the efficiency of interaction via reducing the cost of mental state synchronisation, and thus facilitates collective action within the dyad. There is empirical and theoretical evidence that supports this explanation. For instance, ethnocentricity, i.e., choosing social connections based on ethnic similarity, has been associated with increased efficiency of collective action^20^. A series of mathematical models demonstrated that similarity or “tag” based cooperation can arise autonomously, bypassing the free-rider problem^21,22^.

However, recent empirical results raised questions about the direction of causality: although trait homophily correlates with efficiency in real world collective action, the underlying mechanism might be more complicated. For instance, a longitudinal study of six thousand students^23^ has shown that homophily in academic performance among friends was due not to the friends-will-pull-you-up effect, rather to the rewiring of the friendship networks based on academic performance.

The suggestion that some other dynamics than choosing a partner for effective collective action drives the correlation, pairs well with the observation that many of the particular traits that are taken into account in homophilic friendship choice, such as gender or ethnicity^24,25^, have little to do with effectiveness in collective action. Equally, body similarity is unlikely to correlate with behaviour, despite the fact, that both genetic^26^ and facial^27^ similarity have been shown to trigger positive social affiliation even when relative relatedness was negligible.

Some network similarity traits, e.g., smoking or eating behaviour^28,29^ point to the possibility that homophily has more to do with belonging to a segregated group rather than with collective action efficiency^30–32^. In any case, the focal factors are not necessarily stable: experimental evidence has shown that the traits on which homophilic friendship choice heuristic is based can be shifted using a simple and brief experimental manipulation^33^. This suggests that homophilic friendship choice might have evolved for a different reason than facilitating cooperation in the dyad.

In this paper, I explore an alternative hypothesis: homophily is adaptive because it increases the clustering coefficient of the ego network. If a higher clustering coefficient induces a cooperative stance from the agent’s partners^34^, and thus creates a more trustful network environment, then trait similarity-based friendship choice can be adaptive. For instance, let us assume that a group of individuals are assigned a letter of the alphabet each. Let us focus on an individual with the letter A, who has a choice of possible friends with any letter in the entire range of the alphabet. If she chooses two friends with letters B and C, this choice is unlikely to result in triadic closure as they themselves can choose from the entire range, as well, and hence it is not likely that they will choose each other. However, if A chooses two others who are also As, then, assuming they too are following homophily as choice heuristic, they are more probable to connect to each other. Thus, with a simple similarity-based criterion, a triadic closure is achieved, and the clustering coefficient of all three friends increases.

Empirical evidence supports the idea that people actively increase their clustering coefficients by trying to achieve triadic closure among their social contacts^35^. It may be having a hobby with a social element, joining a club where new friends are also likely to be friends with each other, or playing a team sport in which cooperation is polyadic by design^36–38^. In these, the motivation for using a homophily heuristic as a friend choice strategy is the resulting triadic closure^39,40^.

## Methods

Let *G* denote a set of graphs, each representing kin networks with 500 vertices, generated in a simulated population history with varying fertility^34^. In these binomial kin networks, the edges represent either sibling or first-degree cousin relationships in the following way. The average degree of the graphs in the *G* set varies, determined by the population’s fertility^34^. For any of these kin-only graphs *g* ∈ *G*, let *a* denote the corresponding adjacency matrix, where *a*_*ij*_ = 1 if agents *i* and *j* share two grandparents^34^.

I assume that the desired number of social contacts is uniform among the agents and set at the semi-arbitrary *ν* = 60, such that almost all the agents have fewer relatives than their degree target^34^. To fill the missing social connections, friends are added using the following three methods: (a) homophily only, (b) homophily weighted by graph distance, and (c) random friend choice^34^. (See the Supplementary Information for robustness calculations concerning these assumptions).

### Homophily only

Let us assume that the agents possess a varied trait type, and let *ϕ* denote the value corresponding to the type of an agent, such that

1 $${\phi }_{i} \sim {\rm{U}}(0,100)\forall i=1,\ldots ,n$$

Let *b* denote the adjacency matrix that contains all kin and friend edges. At the beginning of the friendship choice algorithm, let us set *b* = *a*. (For notational simplicity, I drop the algorithm step counter for *b*).

Let *s*_*i*_ denote the number of social contacts for individual *i*:

2 $${s}_{i}\equiv \mathop{\sum }\limits_{j=1}^{n}{a}_{i,j}$$

Step 1: let *X* denote the set of all individuals that have a total number of social contacts lower than *ν*:

3 $$X\equiv \{i\}|{s}_{i} < \upsilon $$

Step 2: select the element, *xi*, from the *X* set that has the lowest number of contacts:

4 $$xi\in X|{s}_{xi}=\,{\rm{\min }}\,{\{{s}_{i}\}}_{i\in X}$$

and select another element, *xj*, that is different to *xi*:

5 $$xj\in X|c({\phi }_{xi},{\phi }_{xj})=\,\min \,{\{c({\phi }_{xi},{\phi }_{j})\}}_{j\in X\backslash xi}$$

where c denotes the type distance:

6 $$c({\phi }_{xi},{\phi }_{xj})=|{\phi }_{xi}-{\phi }_{xj}|$$

Step 3: if they are not connected, create a network edge between *xi* and *xj*:

7 $${b}_{xi,xj}={b}_{xj,xi}=1$$

I repeated the Steps 1–3 until either

8 $${s}_{i}=\upsilon \forall i=1,\ldots ,n$$

or

9 $$\nexists \{xi,xj\}|xi,xj\in X\wedge xi\ne xj\wedge {a}_{xi,xj}=0$$

that is, either all individuals have the limiting number of social contacts, or there are no two elements of *X* left such that they are not connected while their degrees are smaller than the limit.

When this algorithm finishes, the *b* adjacency matrix has its final form, and it defines the full social network graph, *h*. Notice that as *g* is a network that contains the kin relationships, and *h* is a network that contains both kin and friend relationships, *g* is a subgraph of *h*.

### Homophily weighted by graph distance

In the above version of the friend-choice algorithm, the missing social contacts are filled with non-kin friends only on the basis of similarity. This assumes that their choice is unaffected by graph distance. To see the effect of this assumption, let us consider a version in which friends are chosen based on both type similarity and the number of network steps from each other.

For this, I used the Eqs. (1–5) and (7–9) as above, but with (6) replaced by the type distance weighted by the graph distance:

10 $$c({\phi }_{xi},{\phi }_{xj})={({\phi }_{xi}-{\phi }_{xj})}^{2}\cdot {d}_{b}{(xi,xj)}^{0.5}$$

where ${d}_{b}(xi,xj)$ is the graph distance between *xi* and *xj* on the graph defined by the adjacency matrix *b*. (I chose the power factors, arbitrarily set to 2 and 0.5, so that they reflect the assumptions that larger trait distances are disliked with increasing intensity, and changes in graph distance are less important further away from the ego. The results are not qualitatively sensitive to the exact value of these parameters, see the Supplementary Information for robustness calculations).

### Random, homophily independent friend-choice

As a baseline, I include the random friendship choice algorithm^34^, which is the same as (1–4) and (7–9) with (5) replaced by

11 $$xj \sim {\rm{U}}(X\backslash i)$$

Thus, the new connections are entirely random, and both trait similarity and network distance are ignored in friendship choice.

## Results

Let *λ* denote the proportion of social network edges that are non-kin in nature, i.e., *λ* is the ratio of friends to all contacts:

12 $$\lambda =1-\frac{{\Sigma }_{i=1}^{n}{\Sigma }_{j=1}^{n}{a}_{i,j}}{{\Sigma }_{i=1}^{n}{\Sigma }_{j=1}^{n}{b}_{i,j}}$$

Let *χ* denote the average local clustering coefficient of the combined kin and non-kin adjacency matrix, *b*:

13 $$\chi =\frac{{\Sigma }_{i=1}^{n}{\Sigma }_{j=1}^{n}{\Sigma }_{k=1}^{n}{b}_{i,j}{b}_{i,k}{b}_{j,k}}{{n}^{2}\upsilon (\upsilon -1)}$$

that is, *χ* denotes the average number of triadic closures around the agents as a proportion of all possible such triads.

**Figure 1:**
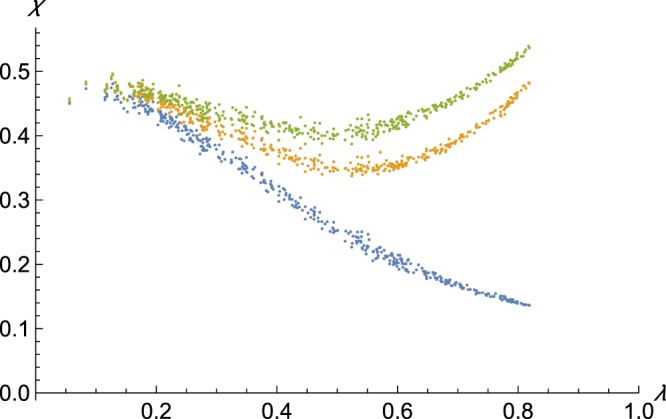
The availability of relatives drives the clustering coefficient. Orange: purely homophily-based friendship choice algorithm. Green: network distance weighted homophily based algorithm. Blue: the baseline random friendship selection. X-axis: the proportion of social contacts that are non-relatives. Y-axis: clustering coefficient.

The results show that the friendship choice algorithm determines the network structure in the following way (Fig. 1). First, the *λ*-dependent *χ* path varies with the friendship choice algorithm such that homophily based-friendship choice raises the average clustering coefficient (Both orange and green are above blue in Fig. 1). Second, homophilic friendship choice is additive with the network proximity-based friendship choice (the green pattern in Fig. 1 is similarly dependent on *λ* as the orange, shifted up).

Third, the *λ*-dependent *χ* path exhibits a transition pattern. When friendship choice is random (blue in Fig. 1), the falling ratio of relatives among the social contacts (moving from left to right on the x-axis in Fig. 1) leads to monotonously decreasing clustering coefficient. When homophily drives friendship choice at least to some extent (orange and green in Fig. 1) then the falling number of available relatives results in a lower clustering coefficient only up to a point. After a trough, the clustering coefficient increases from about mid-point of the x-axis all the way to the right end. Thus, when homophily plays a role in friendship choice, the transition curve is u-shaped.

## Discussion

This paper introduced homophily based friendship choice into a model in which individuals replace missing kin with non-kin friends. The results show that similarity-based friendship choice increases the clustering coefficient, and thus it is an adaptive response to decreasing interconnectedness within the individual’s immediate social network. This is of import as although the clustering coefficient may fall for a number of reasons for any one individual, for an entire society it tends to be associated with demographic processes in which the number of available relatives falls^34^. Urbanisation, falling fertility, migration, war, and epidemics can all result in reduced access to kin, either because relatives are not available, as in urbanisation and migration, or they were never born, as in falling fertility, or have died in wars and epidemics. The consequence of all these demographic processes is a fall in the clustering coefficient that results in increasing incentives for norm violation and evaporating social trust^34^.

### Homophily is adaptive behaviour in increasing the clustering coefficient

It has been suggested in the previous literature that the implementation of law, i.e., an institutionalised third party punishment system is a society-level response to increased norm violations^34^. This paper has shown that homophilic friendship choice can be seen as a parallel, individual-level response. When traditional kin networks break up, the average clustering coefficient falls^34^, which people perceive as feeling lonely, disconnected, surrounded by a social environment in which trust collapses, and norm violating behaviours become frequent^41–43^. To reverse the fall in the clustering coefficient, the individuals’ optimal response is to rely on a friendship choice strategy based on trait similarity. Notice, however, this individually adaptive behaviour may not be a societally optimal response if as a consequence a network cleavage emerges.

### Social identity is a consequence of homophily

The social network literature, which does not have an evolutionary behavioural science-based approach, suggested a causal link from identity to social network homophily^44^: people are raised with socially constructed identities which determine the traits in which people choose each other as social connections. This paper’s logic suggests causality in the opposite direction: first, people agree on the traits on which similarity-based friendship choice can take place. The content of these traits is irrelevant, as long as there is a societal consensus concerning the traits themselves. Using these traits, people build their social networks using trait similarity as a friendship choice heuristic. The consequently high network clustering leads to dense subgroups emerging. The social identity of individuals within such subgroups may then be merely a mental shorthand.

The logic of falling clustering to homophilic friendship choice to segmentation to group identity would explain why the phenomenon of trait-based societal segmentation has emerged historically with urbanisation and falling fertility, and why there is such a wide range of traits along which social identities emerge, despite the similarity of many societal and economic factors in the underlying societies.

### Transition clique-iness

The results suggest a clustering coefficient pattern during demographic processes in a u-shaped curve, predicting that mid-transition societies are particularly prone to a crisis of trust and norm-violation. If this is true, then societies in the middle of demographic change are more likely to exhibit coping behaviours. Shifting from predominantly rural to urban living, a large influx of migration, prolonged war, or deadly epidemics should increase the importance of sub-group membership (e.g., clubs), group signalling behaviours (e.g., special handshakes), and difficult-to-fake markers of origins (e.g., accents). Through the middle of the u-shaped curve, clique-iness becomes normal behaviour, which may fade away once the transition is over if replaced by multi-dimensional trait signalling.

### Fake news

Notice that although the clustering coefficient of a homophily-based friendship network can be as high as for a traditional kin-based one, there remains a crucial difference. While the stability of network edges is high among relatives as these tend to be fixed for life, friendship edges are less certain. Empirically, it has been established that without maintenance via meaningful social interactions, friendship wanes faster than kinship does^15^. Friends, in general, are not chosen for life, these relationships need to be reaffirmed with regularity. To be chosen and re-chosen by their friends, people need to keep signalling their type. This might be particularly pertinent in societies in which others with whom there is a similarity in a number of traits are much more numerous than the personal sociality limit.

In these societies, individuals might feel a need to signal their traits frequently, and in a way that stands out as new. Seasonally changing fashion associated with brands, and competition news about affiliated sport clubs may serve as a source of such flow of markers. Anecdotal observation suggests that it is customary in many societies to start social interaction with a “banter” that refers to news concerning one’s chosen brand (car, clothing, accessory, food etc.) or club (sport, religion, political party etc.) affiliation.

In the past decade, with the rise of social networking apps, a new pathway has opened up for this similarity marker reaffirmation: internet-based news outlets provide a constant flow of possible markers, while social networking apps provide a way of renewing self-assignment to type, by passing on, or “wearing”, the ever newer signals of affiliation. Thus, the fake news phenomenon can be viewed as a side effect of an individual-level coping mechanism responding to decreasing clustering coefficient due to urbanisation, falling fertility, migration, exploited by politically motivated institutions.

In summary, this paper offers a theoretical argument that shows how homophily, i.e., using trait similarity as a friend selection criterion, can increase the clustering coefficient of the social network. Thus, if, as argued in previous literature, higher local connectedness is beneficial for the individual by increasing the cooperative stance of the network partners, achieving this aim using homophily is an adaptive behaviour. Furthermore, the results also suggest that societies that go from a rural, high fertility, low migration state to an urban, low fertility, high migration state are likely to go through a u-shaped transition period in terms of clustering coefficient. If the lower clustering coefficients are associated with increasing norm violations, this theoretical result offers a new explanation for the transition crises in countries that have gone through this shift in recent history.

## Supplementary information

Supplementary Information.

## Data Availability

This is a theoretical paper, there is no data associated with this manuscript.
