# Supplementary material for: Herding Friends in Similarity-Based Architecture of Social Networks
Source: Sci Rep. 2020 Mar 17;10:4859. doi: 10.1038/s41598-020-61330-6 (PMC7078182; doi:10.1038/s41598-020-61330-6)
Supplement: Supplementary file 1 — Supplementary Information. [file 41598_2020_61330_MOESM1_ESM.docx]

**Supplementary Material for**

**Herding Friends in Similarity-Based Architecture of Social Networks**

**Tamas David-Barrett**

To test the results’ robustness towards the model’s assumptions, the following tests were performed.

Summary: These robustness tests show that the main model’s qualitative findings are robust to (1) binary kin assignment, (2) strict kin preference, (3) exact level of degree, (4) uniform degree assumption, and (5) the homophily-network distance weight assignment parameters. The results confirm that the key assumption of the model is that people prefer to populate their social network with kin when these are available.

**Robustness test 1: binary kin assignment and strict kin preference**

The main model assumed that kin assignment is binary, i.e., an alter is either kin or not, and that the edge allocation is based on strict kin preference, i.e., when available, kin are always preferred independent of trait similarity.

To see the effect of the relaxation of these two assumptions, let each agent assign a value to all other agents as a weighted average of their relatedness and their trait similarity:

*βr_i,_*_j_+(1*-β)c_i,j_*

where *β* is the weight parameter, *r_i,_*_j_ is the relatedness coefficient between agents *i* and *j*, and *c_i,j_* is the trait type distance between the agents *i* and *j* inversely normalised between 0 and 1 in line with equation (6) of the paper.

These valuations were used to assign partners to each agent such that each agent has connections to the most preferred others, until the agents reached the limiting contact number of 60. Thus, these network edges constituted the social network graph for each level of kin availability corresponding to the elements of G set of graphs and each *β* parameter. For each consequent new graph, the clustering coefficient was calculated (Fig. S1.)

The result show that the main model’s qualitative findings are robust as long as the *β* parameter is high, albeit in the form of a somewhat less pronounced u-shape. In other words, these calculations confirm that the key assumption of the paper’s model (and that of David-Barrett 2019) is that people in general prefer to populate their social network with relatives. As long as this assumption is present, neither the uniform kin assignment assumption nor the assumption of the model that kin is assigned before friends are, affect the qualitative results of the paper.


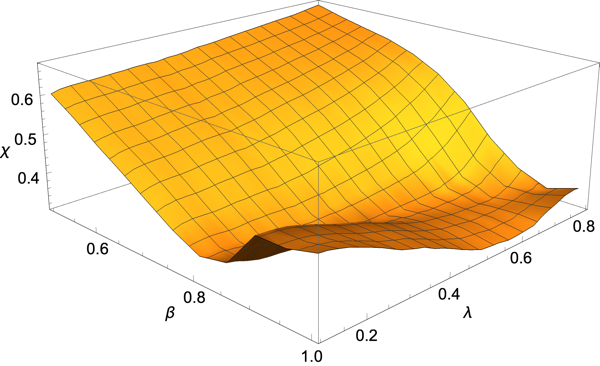


Fig. S1. Robustness test of the assumptions that (a) uniform kin assignment, i.e., that an alter is either kin or not, by allowing the relatedness coefficient take the place of the model’s simpler binary values; and (b) and kin is assigned before friends are, by allowing a combined relatedness and homophily valuation determine the social network edges. *β* is the weight between relatedness coefficient and trait similarity, *λ* is the share of friends in the corresponding graph element of G, and *χ* is the clustering coefficient.

**Robustness test 2: variation in degree**

The paper assumed that each agent’s network degree was identical: 60. To test the finding’s robustness to this assumption, two alternative cases were drawn up in which the agents’ target degrees were uniformly distributed between:

1. 49 and 51
2. 40 and 60

Using these limits, the *h* graphs calculated in the paper were modified, and for each, the clustering coefficient was calculated (Fig. S2, Table S1).

Comparing these results to the paper’s Fig.1, shows that neither the lower mean degree (47.7 and 46.7 respectively, vs. the main model’s 59.8), nor the higher standard deviation (2.5 and 5.2 respectively, vs. the main model’s 0.2) have any effect on the qualitative results of the paper.

Fig. S2. The replication of the paper’s Fig.1, with lower level of average degree and (a) low level of degree variation, and (b) high level of degree variation. Orange: purely homophily-based friendship choice algorithm. Blue: network distance weighted homophily based algorithm. Green: the baseline random friendship selection. X-axis: the proportion of social contacts that are non-relatives. Y-axis: clustering coefficient.

Table S1. Descriptive statistics for the average degree in the original graphs, i.e., the *h* graphs calculated in the paper, as well as the two sets of test graphs: small and high degree variation

**Robustness test 3: network affiliative homophily parameters**

To calculate the weight assigned to the trait similarity vs. the network distance, in equation (10) the paper used somewhat arbitrary parameters that were justified in terms of functional form, but not in terms of the actual values.

To test the robustness of the results, three alternative pairs of parameters were added to the one used in the paper, and the results recalculated using every 20^th^ *h* graph for the network affiliative case. The results show that the qualitative findings are robust to the exact value of the parameters used (Fig. S3).

Fig. S3. The recalculation of the network distance weighted homophily case (the blue dots in Fig. 1), using the varied power parameters. Panel (a): 1 and 1; panel (b): 1.5 and 0.75; panel (c): 2 and 0.5, i.e., the same as in the paper; and panel (d): 4 and 0.25.
